# Stakeholder views of breastfeeding education in schools: a systematic mixed studies review of the literature

**DOI:** 10.1186/s13006-017-0106-0

**Published:** 2017-03-27

**Authors:** Nicola Singletary, Ellen Chetwynd, L. Suzanne Goodell, April Fogleman

**Affiliations:** Department of Food, Bioprocessing, and Nutrition Sciences, North Carolina State University, Box 7624, Raleigh, NC 27695-7624 USA

**Keywords:** Breastfeeding, Infant feeding, Education, Schools, Stakeholders, Students, Teachers

## Abstract

**Background:**

Breastfeeding provides numerous health benefits for mothers and infants, but worldwide breastfeeding rates fall below recommendations. As part of efforts to increase breastfeeding initiation and duration, the World Health Organization and UNICEF UK recommend educational interventions to increase awareness and positive attitudes towards breastfeeding beginning during the school years. Breastfeeding education in the school setting offers the opportunity to improve the knowledge base, address misconceptions, and positively influence beliefs and attitudes for students from a wide range of socioeconomic and cultural backgrounds. The purpose of this paper is to present a comprehensive narrative review of the literature regarding student and teacher (stakeholder) views of breastfeeding and breastfeeding education programs in schools to inform future research in the area.

**Methods:**

Articles were located through a systematic search of online databases and journals using the following keywords in various combinations: (1) breastfeeding, lactation, breast-feeding, “bottle feeding”, “infant feeding” (2) student, educator, teacher, “school administrator” and (3) schools, “secondary education”, “primary education”, “K-12”, “high school”, “middle school”, “elementary school”, education, adolescents, curriculum, and a manual search of article references. Studies were screened for inclusion against specific criteria and included papers were assessed using the Mixed Methods Appraisal Tool (MMAT).

**Results:**

This review suggests that adolescents have a deficit in breastfeeding knowledge and express negative conceptions about breastfeeding. Breastfeeding is being discussed in some school environments, but the extent of lessons and the specific messages that teachers communicate have not been explored. Students appear to be interested in receiving more information about breastfeeding, especially if delivered by health professionals or breastfeeding mothers. The majority of teachers are supportive of incorporating breastfeeding education in family and consumer sciences, sexual education, and health classes; however, time constraints and limited knowledge of infant feeding recommendations may be barriers to implementation of appropriate lesson plans.

**Conclusions:**

Students generally support and are receptive to breastfeeding education; however, research on educator attitudes, knowledge, and experiences are necessary for appropriate implementation of breastfeeding education in varying school settings around the world.

## Background

The health benefits of breastfeeding for both mother and baby are well established [1–3]. The World Health Organization (WHO) and the American Academy of Pediatrics (AAP) recommend exclusive breastfeeding for the first 6 months of life, with continued breastfeeding into and beyond the second year [1, 4]. However, global rates for initiation and duration of breastfeeding fall below these recommendations [3].

In an attempt to increase breastfeeding initiation and duration, global and national policy documents advocate for increased breastfeeding support in our society [4–6]. In the United States, *The Surgeon General’s Call to Action to Support Breastfeeding* recommends education to help increase knowledge, skills, and positive attitudes regarding breastfeeding, emphasizing appropriate support of the mother from her family, health care providers, and community [6]. Likewise, the World Health Organization endorses strategies that allow parents to make informed decisions about infant feeding through the use of evidence-based educational materials that are designed without commercial influence [4]. In particular, this document calls for schools and programs that work with children and adolescents to provide education that promotes awareness and positive attitudes towards breastfeeding as part of the general curriculum [4]. The UNICEF UK Baby Friendly Initiative also recommends breastfeeding education in schools for both male and female students [5]. According to this initiative, providing school-aged children with information about breastfeeding would enable them to make informed choices about infant feeding when they become parents [5].

Women make the decision to breastfeed or formula feed either before or during the early weeks of pregnancy and maintain those decisions throughout their pregnancy [7]. Furthermore, many children and adolescents have already considered infant feeding choices for when they become parents [8–11]. Based on these early decisions regarding infant feeding, efforts to promote breastfeeding during the prenatal or immediate postpartum period may not be effective. Introducing breastfeeding education in the school setting presents a unique opportunity to engage both male and female students from a variety of socioeconomic and cultural backgrounds early in their decision-making process.

In light of the global focus on infant feeding, educational initiatives for school-aged children have the potential to normalize breastfeeding for all segments of the population; however, successful interventions require enabling conditions. Understanding the views and knowledge of stakeholders (educators and students) directly involved with targeted school systems is a critical first step before designing and implementing educational interventions and policies that support breastfeeding. The purpose of this article is a systematic review of the quantitative and qualitative research in order to understand stakeholder knowledge regarding breastfeeding and attitudes towards breastfeeding education in schools. The results of the review are organized as a narrative synthesis discussing the research for each stakeholder group: teachers and students.

## Methods

### Search strategy

A librarian-assisted literature search was conducted by the first author using three online databases: Web of Science, EBSCOhost Research Databases, and PubMed. The last search was conducted on October 7, 2016. Relevant search terms included the following: (1) breastfeeding, lactation, breast-feeding, “bottle feeding”, “infant feeding” (2) student, educator, teacher, “school administrator” and (3) schools, “secondary education”, “primary education”, “K-12”, “high school”, “middle school”, “elementary school”, education, adolescents, curriculum. Additional articles were located using a manual search of the references of studies from the database search, articles that were suggested electronically during the search online, and online archives of journals related to human lactation and health education.

### Screening strategy

During the first phase of screening, the titles and abstracts of the studies identified through the search were screened for inclusion criteria. To be included, articles had to (1) address stakeholder views of breastfeeding or breastfeeding education in schools, (2) be published between 1990 and October 2016, 3) be published in peer-reviewed journals, (4) present original research, and (5) have full text in English. There were no restrictions on the country of study. During the second phase of screening, full text studies were retrieved and assessed according to the same inclusion criteria by two independent reviewers (NS and EC). Disagreements were discussed until consensus was reached.

### Search outcome

**Fig. 1:**
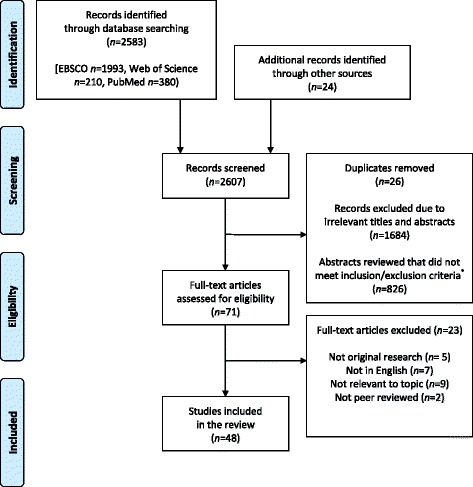
PRISMA (Preferred Reporting Items for Systematic Reviews and Meta-Analyses) flow diagram of literature search

The database search identified 2583 hits (1993 EBSCO, 210 Web of Science, 380 PubMed) and hand searching identified an additional 24 records. After screening the titles and abstracts for inclusion criteria and removing duplicates, 71 articles remained. Full-text examination of the articles left 48 articles for inclusion in this review Fig. 1). Of the 48 articles included, 39 were quantitative studies, one was qualitative and eight were mixed methods studies. Due to the mixed nature of the studies, a mixed studies systematic review process was chosen for this review. This type of review is ideal for synthesizing research of varying methodologies while remaining sensitive to the context of the research across complex fields [12].

### Quality appraisal of studies

The Mixed Methods Appraisal Tool (MMAT) was used for quality assessment of the articles [13]. This quality appraisal tool has been tested for validity, efficiency, and reliability for use with qualitative, quantitative, and mixed methods studies [12]. Two independent reviewers (NS and EC) appraised each study and disagreements were discussed until consensus was reached (Tables 1, 2 and 3).

**Table 1:** Summary, Quality Assessment, and Study Design of Teacher Articles Reviewed

		Study population		Study quality	Questionnaire	
First author & year	Country	N (% female)	Age or class type	Type (MMAT rating)	Designed by & setting	Topics covered
Al-Binali [17], 2012	Saudi Arabia	384 (100)	61.1% primary teacher, 23.2% intermediate school teacher, 12.8% high school teacher, all teachers parent to a child < 5yo	Quantitative descriptive (4.1, 4.2, 4.3. 4.4)	Researcher designed, self-administered in the classroom	Knowledge Attitude Intention Education in schools
Arif [18], 2002	Pakistan	375 (100)	125 low SES 125 mod SES 125 high SES	Quantitative descriptive (4.1, 4.2, 4.3. 4.4)	Researcher designed questionnaire, self-administered in the presence of researcher	Knowledge Attitude
Egbuonu [14], 2004	Nigeria	84 (not given)	Home economics teachers	Quantitative descriptive (4.1, 4.4)	Researcher designed questionnaire at National Diversification and Lactation Management workshop for home economics teachers	Knowledge
Kapil [20], 1992	India	62 (100)	Female married teachers	Quantitative descriptive (4.1, 4.4)	Researcher designed questionnaire, read out loud and explained collectively	Knowledge Attitude
Parrilla Rodriguez [15], 2001	Puerto Rico	125 (89.6)	Health teachers	Quantitative descriptive (4.1, 4.2, 4.3. 4.4)	Researcher designed questionnaire based on literature, self-administered at the beginning of breastfeeding workshop	Knowledge Attitude Personal experience Public breastfeeding
Singh [16], 1990	India	100 (100)	Recently graduated teachers	Quantitative descriptive (4.4)	Researcher designed questionnaire, self-administered	Knowledge Attitude
Spear [21], 2010	United States	107 (79)	53.3% teachers, 1.9% guidance counselor, 44.9% nurses from elementary, middle, and high school	Mixed methods (1.2) (4.1, 4.2, 4.3, 4.4) (5.1, 5.2)	Researcher designed questionnaire modified from previous study [43], self-administered through place of employment	Attitude Intention Breast milk fed Public breastfeeding Sources of information Education in schools
Veghari [19], 2011	Northern Iran	745 (46)	Primary school teachers	Quantitative descriptive (4.1, 4.2, 4.4)	Researcher designed questionnaire, unknown administration	Knowledge

The Mixed Method Appraisal Tool (MMAT) was used to evaluate the articles in the review. Listed are the criteria met for each study based on its methodological category. See the MMAT appraisal checklist for full explanation of criteria [13]

• Qualitative (1.1 to 1.4)

• Quantitative randomized controlled (2.1 to 2.4)

• Quantitative non-randomized (3.1 to 3.4)

• Quantitative descriptive (4.1 to 4.4)

• Mixed methods studies include both the qualitative criteria and the appropriate quantitative criteria in addition to criteria specific for mixed methods studies (5.1 to 5.3)

*Knowledge*: Specific topics including mechanics or immune benefits of nursing, with correct or incorrect answers

*Attitude*: Attitudes about ease, acceptance, beliefs about benefits, feelings about breastfeeding or breastfeeding education

*Intention*: Goals for future breastfeeding or support for breastfeeding partner

*Breast milk fed*: Participant nursed as infant

*Personal exposure*: Whether participant has seen others feed a baby by bottle or breast

*In public*: Attitude about breastfeeding in public settings

**Table 2:** Summary, Quality Assessment, and Study Design of Primary School Articles Reviewed

		Study population		Study quality	Questionnaire	
First author & year	Country	N (% female)	Age (years) or class type	Type (MMAT rating)	Designed by & setting	Topics covered
Angell [23], 2011	England	56 (48)	Age 5 to 11	Qualitative (1.1, 1.2, 1.3, 1.4)	Researcher led draw, write, tell. Group pictures, individual child-led description of pictures	Attitude
Bottaro [47], 2009	Brazil	564 (50)	Age 9 to 17 (5th grade)	Quantitative randomized (2.1, 2.2, 2.3, 2.4)	Researcher designed questionnaire. Self-administered in classroom in the presence of researcher / some questions as vignettes	Knowledge Attitude Breast milk fed Personal exposure Public breastfeeding
Costa [46], 2006	Brazil	32 (56)	Age 7 to 12	Quantitative non-randomized (3.3, 3.4)	Researcher designed questionnaire. Self-administered	Knowledge Attitude Intention
Fujimori [8], 2008	Brazil	503 (54)	Mean age 11.7	Quantitative non-randomized (3.1, 3.2, 3.3, 3.4)	Researcher designed questionnaire. Self-administered, teacher supervised	Knowledge Personal exposure Sources of information Education in schools
Galvao [9], 2011	Portugal	1078 (46)	Age 7 to 12	Quantitative descriptive (4.1, 4.2, 4.3, 4.4)	Researcher designed questionnaire. Self-administered, teacher supervised, in classroom	Attitude Intention Breast milk fed Personal exposure Sources of information
Russell [24], 2004	Scotland	23 (61)	Age 6	Mixed methods (1.1, 1.2, 1.3, 1.4) (4.1, 4.2, 4.3,4.4) (5.1, 5.2)	Researcher designed questionnaire to parents administered at home. Focus groups with children led by two experienced facilitators	Knowledge Attitude Breast milk fed Personal exposure Public breastfeeding Sources of information

The Mixed Method Appraisal Tool (MMAT) was used to evaluate the articles in the review. Listed are the criteria met for each study based on its methodological category. See the MMAT appraisal checklist for full explanation of criteria [13]

• Qualitative (1.1 to 1.4)

• Quantitative randomized controlled (2.1 to 2.4)

• Quantitative non-randomized (3.1 to 3.4)

• Quantitative descriptive (4.1 to 4.4)

• Mixed methods studies include both the qualitative criteria and the appropriate quantitative criteria in addition to criteria specific for mixed methods studies (5.1 to 5.3)

*Knowledge*: Specific topics including mechanics or immune benefits of nursing, with correct or incorrect answers

*Attitude*: Attitudes about ease, acceptance, beliefs about benefits, feelings about breastfeeding or breastfeeding education

*Intention*: Goals for future breastfeeding or support for breastfeeding partner

*Breast milk fed*: Participant nursed as infant

*Personal exposure*: Whether participant has seen others feed a baby by bottle or breast

*In public*: Attitude about breastfeeding in public settings

*Sources of information*: Where participant learned about breastfeeding

*Education in schools*: Whether participant desires more education or feels education should be offered in school

**Table 3:** Summary, Quality Assessment, and Study Design of Secondary School Articles Reviewed

		Study population		Study quality	Questionnaire	
First author & year	Country	N (% female)	Age or class type	Type (MMAT rating)	Designed by & setting	Topics covered
Alnasir [26], 1992	Bahrain	100 (100)	Age 15 to 17	Quantitative descriptive (4.1, 4.2, 4.4)	Researcher designed questionnaire and individual interviews	Knowledge Attitude Intention Breast milk fed Personal exposure
Bailey [55], 2007	England	92 (100)	Age 14 to 15	Quantitative non-randomized (3.1, 3.2, 3.3, 3.4)	Iowa Infant Feeding Attitude Scale and researcher designed questionnaire. Self-administered in a classroom in the presence of researcher	Attitude
Bomba [45], 2009	United States	510 (76)	University of Mississippi family and consumer science class students	Quantitative descriptive (4.3)	Researcher designed questionnaire, based on literature. Self-administered in classroom	Knowledge Public breastfeeding Sources of information
Connolly [25], 1998	Ireland	177 (35)	Age 16 to 19	Mixed methods (1.1, 1.2) (4.1, 4.2, 4.3, 4.4) (5.1, 5.2)	Researcher designed questionnaire based on TRA and TPB. Self-administered	Knowledge Attitude Intention Personal exposure Sources of information
Forrester [38], 1997	United States	590 (69)	346 age 13 to 19 (high school), 244 age 17 to 43 (college)	Quantitative descriptive (4.1, 4.2, 4.3)	Researcher designed Questionnaire. Self-administered, teacher supervised	Attitude Intention Breast milk fed Public breastfeeding Sources of information Education in schools
Frew [48], 2005	United States	37 (unknown)	High school students (age not given)	Quantitative descriptive (4.1, 4.2, 4.3)	Questionnaire adapted from a K-12 breastfeeding curriculum. Self-administered in school auditorium prior to presentation on breastfeeding	Knowledge Attitude Public breastfeeding
Gale [10], 2013	England	81 (60)	Age 13 to 15	Quantitative descriptive (4.1, 4.3)	Researcher designed questionnaire. Self-administered in presence of health teacher. Participants voluntarily attended a session during school break.	Knowledge Attitude Intention Personal exposure Sources of information Education in schools
Giles [36], 2007	Northern Ireland	Qualitative: 48 (54) Quantitative: 121 (50)	Age 13 to 14	Mixed methods (1.1, 1.2, 1.3, 1.4) (4.1, 4.2, 4.3, 4.4) (5.1, 5.2, 5.3)	Researcher designed and led focus groups based on TPB / Researcher designed questionnaire based on TPB and modal beliefs elicited in semi-formatted focus groups. Self-administered in classroom in presence of researcher	Knowledge Attitude Intention Personal exposure Public breastfeeding
Giles [22], 2010	Northern Ireland	2021 (58)	Age 13 to 14	Quantitative descriptive (4.1, 4.2, 4.3)	Researcher designed questionnaire based on TPB and modal beliefs elicited in semi-formatted focus groups. Self-administered in the presence of the researcher	Knowledge Attitude Intention Personal exposure Public breastfeeding Sources of information
Giles [54], 2015	Northern Ireland	Qualitative: 48 (54) Quantitative: 2021 (58)	Age 13 to 14	Mixed methods (1.1, 1.2, 1.3, 1.4) (4.1, 4.2, 4.3, 4.4) (5.1, 5.2, 5.3)	Researcher designed and led semi-formatted focus groups based on TPB. Researcher designed questionnaire based on TPB and modal beliefs elicited in focus groups. Self-administered in the presence of the researcher	Knowledge Attitude Intention Personal exposure Public breastfeeding Sources of information
Giles [49], 2014	Northern Ireland	698 (not given)	Age 13 to 14	Quantitative randomized (2.1, 2.2, 2.3, 2.4)	Researcher designed and led semi formatted focus groups based on TPB. Researcher designed questionnaire based on TPB and modal beliefs elicited in focus groups. Self-administered in the presence of the researcher	Knowledge Attitude Intention Personal exposure Public breastfeeding Sources of information
Gostling [27], 2003	England	217 (62)	Age 13 to 15	Quantitative descriptive (4.1, 4.2, 4.4)	Researcher designed online questionnaire administered by technology teacher	Attitude Intention Breast milk fed Personal exposure Public breastfeeding
Goulet [28], 2003	Canada	439 (54)	Age 12 to 19	Quantitative descriptive (4.1, 4.2, 4.3, 4.4)	Researcher designed questionnaire (based on TRA). Self-administered during class time	Attitude Breast milk fed Personal exposure Public breastfeeding
Greene [40], 2003	Northern Ireland	419 (57)	Age 14 to 18	Quantitative descriptive (4.1, 4.2, 4.3, 4.4)	Researcher designed questionnaire (based on focus groups with pregnant women). Self-administered in school	Attitude Public breastfeeding Sources of information Education in schools
Hadley [35], 2008	Ethiopia	2077 (49)	Age 13 to 17	Quantitative descriptive (4.1, 4.2, 4.3, 4.4)	Secondary analysis of survey data based on the WHO Infant and Young Child Feeding Behavior Model. Interview by researcher in home.	Knowledge Intention
Ho [31], 2014	Taiwan	1319 (61)	Age 15 to 17	Quantitative descriptive (4.1, 4.2, 4.3, 4.4)	Iowa Infant Feeding Attitude Scale. Self-administered, distributed to teachers	Attitude Intention Personal exposure Sources of information Education in schools
Ho [56], 2016	Taiwan	204 (100)	Age 16+, mean 16.9	Quantitative non-randomized (3.2, 3.3, 3.4)	Iowa Infant Feeding Attitude Scale and researcher designed Breastfeeding Knowledge Scale. Self-administered in the classroom in the presence of the researcher	Knowledge Attitude Breast milk fed Personal exposure Sources of information
Kapil [33], 1990	India	152 (100)	Adolescence (age not described)	Quantitative descriptive (4.1, 4.2, 4.4)	Researcher designed questionnaire. Read out loud and explained in classroom	Knowledge Attitude
Kim [50], 1998	Korea	412 (100)	Age 16	Quantitative non-randomized (3.1, 3.2, 3.3, 3.4)	Adapted from previous questionnaires. Self-administered in the presence of the researcher	Attitude Intention Personal exposure
Leffler [32], 2000	United States	100 (100)	Age 14 to 19	Quantitative descriptive (4.1, 4.2, 4.3, 4.4)	Researcher designed questionnaire. Self-administered after individual recruitment in cafeteria or supervised study periods	Attitude Intention Breast milk fed Personal exposure Public breastfeeding Education in schools
Lockey [51], 2003	England	101 (48)	Age 13 to 15	Mixed methods (1.1, 1.3) (4.2, 4.4) (5.2)	Open dialogue in focus groups	Knowledge Attitude
Martens [52], 2001	Canada	45 (53)	Mean age 13	Quantitative randomized (2.1, 2.3)	Questionnaire adapted from previous work with same population. Self-administered in classroom	Attitude Intention Breast milk fed Personal exposure Public breastfeeding
Nkanginieme [29], 1993	Nigeria	824 (62)	Age 15 to 20	Quantitative descriptive (4.1, 4.2, 4.4)	Researcher designed questionnaire, self-administered in classroom	Knowledge Attitude Intention Breast milk fed Personal exposure Public breastfeeding Sources of information
November [57], 2013	England		Age 13 to 16	Mixed methods (1.1, 1.2, 1.3) (3.1, 3.2, 3.3, 3.4) (5.1)	Iowa Infant Feeding Attitude Scale. Anonymous written feedback. Self-administered in classroom. Qualitative responses at end of session on post-it notes in the presence of the researcher	Attitude Intention Breast milk fed
Ojofeitimi [37], 2001	Nigeria	34 (100)	Age 15 to 19	Quantitative descriptive (4.1, 4.4)	Researcher designed questionnaire, self-administered in the presence of researcher	Knowledge Attitude
Purtell [11], 1994	England	40 (100)	Age 16 to 17	Quantitative descriptive (4.3, 4.4)	Researcher designed questionnaire, self-administered, half with the researcher, half without.	Attitude Intention Breast milk fed Personal exposure Education in schools
Rasheed [30], 1994	Saudi Arabia	589 (100)	Age 16 to 19	Quantitative descriptive (4.1, 4.2, 4.3, 4.4)	Researcher designed questionnaire, self-administered	Knowledge Attitude Intention
Seidel [39], 2013	United States	107 (77)	Age 14 to 19	Quantitative non-randomized (3.1, 3.2)	Researcher designed questionnaire based on literature and TPB. Self-administered in the presence of researcher	Knowledge Attitude Intention
Spear [43], 2007	United States	515 (65)	College students in general education and nursing courses	Mixed methods (1.2) (4.1, 4.3, 4.4) (5.2)	Researcher designed questionnaire, self-administered in presence of researcher	Attitude Intention Breast milk fed Public breastfeeding Sources of information Education in schools
Swanson [34], 2006	Scotland	229 (54)	Ages 11 to 18	Quantitative descriptive (4.1, 4.2, 4.3, 4.4)	Researcher designed questionnaire based on other studies using TRA/TPB. Self-administered in presence of researcher	Knowledge Attitude Intention Breast milk fed Personal exposure Public breastfeeding
Tjiang [44], 2001	Australia	136 (100)	University students using Indonesian student association mailing list	Quantitative descriptive (4.1, 4.2, 4.3)	Researcher designed- questionnaire generated from related literature. Self-administered postal survey	Knowledge Attitude Breast milk fed Personal exposure Sources of information
Walsh [53], 2008	Canada	121 (66)	Age 15 to 19	Quantitative non-randomized (3.2, 3.3, 3.4)	Researcher designed questionnaire, self-administered in presence of researcher	Knowledge Intention Breast milk fed Personal exposure Sources of information
Yeo [41], 1994	Japan and United States	329 (100) 242 Japan, 87 US	Age 16 to 17	Quantitative descriptive (4.1, 4.2, 4.3, 4.4)	Questionnaire designed by another researcher (Berger 1980). Self- administered postal survey	Attitude Intention Breast milk fed Education in schools
Zeller [42], 2016	United States	39 (13)	Age 12 to 13	Quantitative non-randomized (3.2, 3.4)	Questionnaire designed by another researcher (Martens 2001). Self-administered in the presence of the researcher	Attitude Intention Breast milk fed Personal exposure Education in schools

The Mixed Method Appraisal Tool (MMAT) was used to evaluate the articles in the review. Listed are the criteria met for each study based on its methodological category. See the MMAT appraisal checklist for full explanation of criteria [13]

• Qualitative (1.1 to 1.4)

• Quantitative randomized controlled (2.1 to 2.4)

• Quantitative non-randomized (3.1 to 3.4)

• Quantitative descriptive (4.1 to 4.4)

• Mixed methods studies include both the qualitative criteria and the appropriate quantitative criteria in addition to criteria specific for mixed methods studies (5.1 to 5.3)

*Knowledge*: Specific topics including mechanics or immune benefits of nursing, with correct or incorrect answers

*Attitude*: Attitudes about ease, acceptance, beliefs about benefits, feelings about breastfeeding or breastfeeding education

*Intention*: Goals for future breastfeeding or support for breastfeeding partner

*Breast milk fed*: Participant nursed as infant

*Personal exposure*: Whether participant has seen others feed a baby by bottle or breast

*In public*: Attitude about breastfeeding in public settings

*TRA* Theory of Reasoned Action; *TPB* Theory of Planned Behavior

## Results and Discussion

### Teacher knowledge of breastfeeding and views regarding breastfeeding education

The willingness and ability of teachers to incorporate infant feeding education in their classrooms is a critical component of research on school breastfeeding education program development and implementation. Seven studies examined teachers’ knowledge of breastfeeding [14–20] and two investigated their views on incorporating breastfeeding education into schools [14, 21].

Teachers’ knowledge of and attitudes toward infant feeding recommendations and practices have implications on their willingness and ability to present breastfeeding education to their students. The research that has been done indicates that teachers have a basic knowledge of the benefits of breastfeeding but are not aware of or have misconceptions about specific infant feeding recommendations. In 2004, a study was conducted in Nigeria of 84 home economics teachers. The study was designed to assess teacher awareness of breastfeeding and the Baby Friendly Initiative (BFI). Researchers found that 70.2% of teachers surveyed knew that human milk can prevent malnutrition and 53.6% agreed that breastfeeding should continue for at least 2 years. Beyond this knowledge, the teachers’ understanding was low in regards to the concepts of the BFI, the benefits of colostrum, feeding on demand, and protection of breastfeeding by the law [14]. Similarly, in a 1990 study of infant feeding knowledge among 100 newly graduated Indian teachers, researchers found that the majority identified human milk as the best way to feed infants and that babies should be breastfed for over 1 year. Of those surveyed, 77% knew that breastfeeding is beneficial for the mother, and 90% recognized that breastfeeding is important in mother-child bonding. However, many had misconceptions about infant feeding best practices, including 61% agreeing that first feeds other than colostrum are appropriate and 100% believing that milk needs to be diluted with water so it is not too heavy for the infant’s stomach [16]. In Pakistan, the breastfeeding knowledge of 375 female school teachers from different socioeconomic areas was assessed. The majority of teachers in each socioeconomic group knew that breastfeeding on demand is preferred and that mothers need additional food when breastfeeding, but less than one fourth knew that babies should only be given breast milk for the first 6 months of life and that breastfeeding should be continued to 18–24 months of life [18]. Research with 384 female teachers who had a child 5 years of age or less in Saudi Arabia indicated that 89.3% knew the benefits of colostrum, but only 28% identified that a child should receive only breast milk for the first 6 months of life [17]. In northern Iran, 745 male and female primary school teachers completed a questionnaire about infant feeding. Overall, 81.6% agreed that breastfeeding is beneficial during the first 6 months of life, but 17.6% also considered other milk or milk substitutes appropriate [19]. Furthermore, a study in 2001 of 125 Puerto Rican health teachers showed that 96.7% agreed that breastfeeding helps establish a bond between mother and child, 98.4% agreed it protects the baby against infections, and 98.4% agreed it provides the best nutrition for the baby. However, 60.3% agreed that mothers must follow a specific diet when breastfeeding and 36% thought that breastfeeding should be alternated with formula [15]. These studies indicate that teachers may have generally positive attitudes towards breastfeeding and knowledge of the basic benefits of breast milk feeding, but they have limited knowledge of and incorrect beliefs about specific infant feeding recommendations.

Previous research on teachers’ attitudes toward including breastfeeding education in schools indicates that the majority of teachers are supportive of including breastfeeding education in the classroom [14, 21], but they have limited time to include this material in the curriculum [21]. The Nigerian study by Egbuonu et al. concluded that the majority of the 84 home economics teachers surveyed were supportive of teaching about the BFI in primary, secondary, and tertiary education [14]. Similar research in the United States conducted in 2010 showed that 86.9% of the 107 teachers and school nurses surveyed thought that the benefits of breastfeeding should be incorporated into high school curricula, and 57.9% believed the topic should be taught at the middle school level. The teachers and school nurses identified that the following classes would be the most appropriate subjects in which to incorporate breastfeeding content: 34.6% family life, 11.2% health, 11.2% sex education, and 8.4% science. In addition, the majority of those surveyed agreed that it is important to promote a breastfeeding culture in the United States and that breastfeeding in public is acceptable. While the majority agreed that breastfeeding education should be included in the curriculum, only 15.9% were teaching about the benefits of breastfeeding, 5.6% at the middle school level and 10.3% at the high school level. Reasons cited for not teaching about breastfeeding include limited time and lack of breastfeeding education in the required curriculum. A minority of teachers and nurses believed that breastfeeding should not be taught in the classroom (13% at the high school level and 42% at middle school level) despite agreeing that all mothers should hear about the distinctive benefits of breastfeeding from their health care providers. Open response comments ranged from “Breastfeeding should be strongly encouraged” to “Students should be educated about breastfeeding, but breastfeeding should not be endorsed” (pg. 142) [21]. Although these two studies covered a limited geographical area, they suggest that teachers may be willing to incorporate the benefits of breastfeeding into family and consumer science, health, or science classes if it is part of the required curriculum or could easily be integrated with existing curriculum goals and lessons. Despite research that many teachers support including breastfeeding education in the general curriculum, some teachers and administrators feel that breastfeeding content is only suitable for female students [22] and are concerned that breastfeeding education is a sensitive subject closely linked to teen pregnancy [21, 22]. In addition, researchers have met reluctance from school administrators when attempting to conduct research on breastfeeding education within schools. Administrators cited concerns regarding the appropriateness of the subject matter for the age or gender of the students [21].

### Student knowledge of breastfeeding and views regarding breastfeeding education

Understanding the views of students regarding breastfeeding and breastfeeding education is an important part of designing appropriately targeted educational interventions. With this knowledge, educators can develop and implement lessons and curricula that are student-centered and focus on the areas of greatest need and potential impact. Due to the differences in school age classification internationally, for this review we define primary as age 11 or younger and secondary as ages 12–19 unless otherwise indicated in Tables 2 and 3.

In the primary grades, some girls and boys are aware of breastfeeding through exposure to infant feeding in their family or community, but many have already internalized bottle feeding and formula as the norm for infant feeding [8, 9, 23, 24]. The results of research on the attitudes toward breastfeeding for secondary male and female students are mixed. Some studies demonstrate positive attitudes towards breastfeeding [25–30], while other results are neutral or negative [25, 31]. Even students with positive attitudes towards breastfeeding can lack specific knowledge of breastfeeding and infant feeding recommendations [8, 9, 22, 25, 29, 30, 32–37]. Many students know that breastfeeding has health benefits for babies [9–11, 25, 26, 28, 33, 36, 38]; however, most children are not aware of the specific benefits of breastfeeding for the infant or for the mother [8–11, 22, 33, 34, 36, 39]. Furthermore, students have misconceptions that breastfeeding can have a negative impact on the mother’s health and lifestyle [9, 10, 33].

Primary [23, 24] and secondary [10, 11, 27, 31, 38, 40, 41] school children are receptive to breastfeeding education in schools. Secondary school students also recognize that education has the potential to increase awareness and knowledge of breastfeeding and help normalize breastfeeding [10, 27, 38, 42]. Inclusion of breastfeeding education in the school setting is limited. In a survey of 515 United States college students, 36.7% remembered being taught about breastfeeding during high school and only 11% recalled lessons on the topic during middle school [43]. Comparatively, only 6.6% of 136 female students in Indonesia recalled learning about breastfeeding in school [44]. In similar research in the United States, England, Northern Ireland, and Taiwan, approximately one fourth of secondary students reported receiving information about breastfeeding in high school [10, 31, 38, 40, 45]. In contrast, 52% of girls in Bahrain and 48.6% of students in Nigeria recalled being taught about infant feeding in school [26, 29].

There is an emerging body of work that shows that interventions with children [8, 46, 47] and adolescents [39, 42, 48–57] in the school environment have the potential to positively affect their breastfeeding attitudes and knowledge. In research by Greene et al. in Northern Ireland, 76% of secondary students surveyed agreed that information about breastfeeding should be part of the main curriculum, with 88% agreeing it should be part of the child development module, 78% part of sex education, and 62% part of home economics [40]. However, some adolescents are unsure whether teachers have adequate knowledge to teach this information and may prefer that lessons be taught by a health care provider or a breastfeeding mother [11, 40].

Surveys of college students in the United States also show support for breastfeeding education at the secondary school level [43, 45]. In a 2007 study by Spear, the majority of the college students surveyed believed the benefits of breastfeeding should be included in the high school curriculum (87.2% agreed or strongly agreed and 12.8% disagreed or strongly disagreed) but only one third thought it should be included in the middle school curriculum (34.9% agreed or strongly agreed and 65.1% disagreed or strongly disagreed) [43]. Another survey of college students showed that they felt breastfeeding education was more acceptable for students at the high school level compared to the middle school level and more so for girls than boys (78% high school girls, 42% high school boys, 32% middle school girls and 15% middle school boys). In addition, 45% of respondents identified that high school teachers are an important source of infant feeding practices education [45].

## Conclusions

This review provides evidence that primary and secondary school teachers are willing to incorporate infant feeding education into the classroom and many understand the basic benefits of breastfeeding. Teacher support enables the development and implementation of breastfeeding education programs as a vital component of breastfeeding promotion initiatives. Additional research into the attitudes, knowledge, and experiences of educators regarding teaching breastfeeding in schools would add to our understanding of how best to implement lessons and curricula in the future. Areas of study should include teachers’ views on barriers to implementing breastfeeding education in schools, teachers’ comfort with the content and knowledge of breastfeeding, the best way to address gaps in teacher knowledge, and the replication of teacher acceptability of breastfeeding education in a variety of geographic areas.

This review indicates that breastfeeding is being discussed in some school environments, but the research is limited geographically. Furthermore, the extent and specific messages children receive have not been explored. In many cases, students are interested in receiving more information about breastfeeding, especially if it comes from health professionals or breastfeeding mothers. It is imperative that the education children get from school provides unbiased information about current feeding recommendations, enabling them to make informed decisions when they become parents. It is also critical to consider the views of other major stakeholders when designing and implementing educational programs that address breastfeeding. Researchers indicated that they encountered resistance from school administrators, but there is very little research on the views of administrators regarding breastfeeding education in schools.

A limitation of this review is that it does not include research from unpublished dissertations and theses or studies published in languages other than English. Despite this limitation, this review suggests that breastfeeding education in the school setting offers the opportunity to introduce the topic to a wide range of students from a variety of socioeconomic and cultural backgrounds around the world. By introducing the topic in schools we can build knowledge and positive ideas throughout childhood and adolescence that can be carried into adulthood regardless of a persons’ ethnicity, culture, education, or income. Incorporating positive breastfeeding messages as part of the health, science, and family and consumer science curricula in schools will promote a society that is supportive of breastfeeding.

Existing research offers us information that can be used to develop targeted educational programs for primary and secondary schools. These programs should work towards improving awareness of breastfeeding in general and address areas shown as lacking in student knowledge such as the specific benefits of breastfeeding for the mother, the infant, and their relationship. Well-crafted lessons have the potential to increase understanding of the importance of breastfeeding and dispel myths that breastfeeding has a negative impact on the health and well-being of the mother. These programs are needed to address negative attitudes toward breastfeeding and to respond to students’ receptiveness to more information on the subject, enabling them to make informed decisions as they enter adulthood and parenthood. Correcting misconceptions about breastfeeding is beneficial for future parents and public health in general, creating a more accepting and supportive culture for breastfeeding.
